# Evaluation of compressive strength, surface microhardness, solubility and antimicrobial effect of glass ionomer dental cement reinforced with silver doped carbon nanotube fillers

**DOI:** 10.1186/s12903-023-03542-6

**Published:** 2023-10-23

**Authors:** Tamer M. Hamdy

**Affiliations:** Restorative and Dental Materials Department, Oral and Dental Research Institute, National Research Centre (NRC), Giza, 12622 Dokki Egypt

**Keywords:** Dentistry, Glass ionomer, Silver, Carbon nanotubes, Compressive strength, Microhardness, Solubility, Antimicrobial activity

## Abstract

**Background:**

Conventional glass ionomer cements (GICs) are currently the most widely used dental cements due to their chemical bonding into tooth structure, release of fluoride, and ease of manipulation and usage. One of their drawbacks is their low mechanical properties and high solubility. Carbon nanotubes (CNTs) could be utilized in dentistry due to their several potential applications. CNTs can be used as fillers to reinforce polymers or other materials. Additionally, silver (Ag) nanoparticles are highly effective at preventing dental biofilm and enhancing mechanical properties.

**Objectives:**

The aim of the present in vitro study is to evaluate the compressive strength, surface microhardness, solubility, and antimicrobial effect of the conventional GIC reinforced with manual blending of 0.01 wt.% Ag doped CNT fillers.

**Methods:**

The control group was prepared by mixing dental GIC powder with their liquid. The innovatively reinforced dental GIC group was prepared by incorporating 0.01 wt.% Ag doped CNT fillers into the GIC powder prior to liquid mixing. Chemical characterization was performed by XRF. While, physical characterization was done by measuring film thickness and initial setting time.

The compressive strength, surface microhardness, solubility, and antimicrobial effect against *Streptococcus mutans* bacteria using an agar diffusion test were measured. The data was statistically analyzed using independent sample *t*-tests to compare mean values of compressive strength, surface microhardness, solubility, and antimicrobial activity (*p* ≤ 0.05).

**Results:**

The results revealed that innovative reinforced GIC with 0.01 wt.% Ag doped CNT fillers showed higher mean compressive strength, surface microhardness, and antimicrobial effect values than the conventional GIC control group; there was no significant difference between different groups in relation to the solubility test (*P* ≤ 0.05).

**Conclusion:**

The innovatively reinforced GIC with 0.01 wt.% Ag doped CNT fillers had the opportunity to be used as an alternative to conventional GIC dental cements.

## Background

Polymers have widely dental applications [1–4]. Glass ionomer cement (GIC) is an aesthetic restorative material with a self-adhesive property [5, 6]. It is a mixture of fluoro-aluminosilicate glass powder and liquid polyacrylic acid in terms of chemistry [7]. It has been employed in a variety of dental applications, including minimally invasive and atraumatic direct restoratives, liner and bases, pit and fissure sealants, and endodontic sealers [8, 9]. Clinicians prefer to use GICs for long-term temporary restorations, the sandwich approach, luting agent, root caries, and permanent restorations in non-stress bearing areas, also cementation of orthodontic appliances and prosthodontic frameworks on the primary teeth [10–12]. Their properties are generally attractive for these applications and include high aesthetic properties, biocompatibility, fluoride-releasing and remineralization ability, chemical adhesion to the tooth surface, and matching the coefficient of thermal expansion of the natural tooth [13, 14]. Despite these benefits, their main disadvantage is low early mechanical properties in sites subjected to high stresses, and low wear resistance, in addition to their high solubility, which may affect their survival rates when used in load-bearing regions [13]. Film thickness and the initial setting time of the dental cements are two significant physical properties of an important aspect of restorative dentistry [15]. Several attempts were performed to improve the conventional GICs to stun their drawbacks, many studies aimed to enhance the mechanical characteristics of GICs. This was accomplished by adding nanofiller particles to GICs, such as bioactive glass, hydroxyapatite powders, metallic powders, nanoclay, and glass fibers [16–19].

Carbon nanotubes (CNTs) are cylindrical hollow structures consisting of a hexagonal network of carbon atoms at the nanoscale [20]. CNTs can be utilized as fillers to strengthen polymers or other materials, forming metal-based inorganic nanoparticles [21]. Multi-walled CNTs have emerged as superior fillers due to their larger surface area, high loading capacity, and potential to interact with biological membranes [22]. Consequently, their higher mechanical features and enhanced antimicrobial activity suggest that they would be utilized as promising fillers for dental applications [23]. They demonstrated adequate performance in reinforcement due to the load transfer on the interfacial bonding of the reinforced material and the CNTs [24].

Several metals, like silver, copper, gold, titanium, and zinc, have been employed as antimicrobial materials. Each of these metals has unique properties and a range of activities [25]. Silver (Ag) nanoparticles have long been utilized in dentistry due to their antibacterial activity. They are considered an efficient antibacterial filler, have great potential to inhibit biofilm, and also act as strengthening agents [20]. Functionalizing CNTs with other metallic nanoparticles such as gold, palladium, platinum, and silver may be able to produce nanofillers with predictable superior properties [26]. The doping of CNTs with Ag nanoparticles has potential applications as advanced nanocomposites with enhanced mechanical properties and antibacterial potential [22].

The aim of the present study was to evaluate the compressive strength, surface microhardness, solubility, and antimicrobial effect of dental GIC reinforced with Ag doped multi-walled CNT fillers against the untreated GIC (control). The null hypothesis was that the incorporation of 0.01 wt.% Ag doped CNT into conventional GIC would not affect the compressive strength, surface microhardness, solubility, or antimicrobial effect against *Streptococcus mutans* compared to the untreated control group.

## Methods

The present experimental study was approved by the Medical Research Ethical Committee (MREC) of the National Research Centre (NRC), Cairo, Egypt (Reference number: 98072032023).

For this study, a commercially available conventional chemically cured GIC was provided in powder and liquid form: Fuji IX GP Extra (GC Corporation, Tokyo, Japan). Commercial multi-walled CNT doped with 12 wt.% Ag nanoparticle powder was also used (Nanografi Nano Technology, Jena, Germany). The specifications of the Ag-doped CNT were: an average particle length range of 15–25 nm, an average outside diameter less than 50 nm, an average inside diameter of 5 nm, and an average purity of more than 97 wt.%.

### Preparation of the specimens

A total of 80 specimens were used. The specimens were divided into two main groups (*n* = 40/group) based on the type of powder used in the mixing process. The sample size was calculated using the G*Power (version 3.1.9.7) sample size calculator based on means and standard deviations [27]. The estimated sample size for each group was 10. The control group was prepared by mixing the conventional GIC powder with their liquid; the treated group was obtained by hand-mixing of 0.01 wt.% silver-doped CNT fillers with the conventional GIC powder using a mortar and pestle to obtain a homogenous powder. The prepared powder was then mixed with their liquid. The mixing of powder and liquid was done according to the manufacturer’s instructions. The mixed material from each group was placed in custom-made Teflon molds according to each test. A polyester strip was placed to prevent air trapping, and gentle compression was applied to the materials on both sides of the mold by a glass slide. Specimens were removed from the mold after the setting time specified by the manufacturer. The specimens were then examined visually for imperfections.

### Characterization of prepared specimens

Chemical characterization of the control and treated specimens were performed using a non-destructive X-ray fluorescence (XRF) analysis (X-MET3000TXR, Oxford Instruments GmbH Co., Borsigstrasse, Germany) to verify their chemical composition [28].

Physical characterization of the control and treated specimens were assessed by measuring film thickness and setting time. The film thickness of the control and treated specimens was investigated according to the International Standard Organization (ISO) ISO standard 9917–2 instructions [29]. Using an electronic digital caliper (Digital Vernier Caliper, Mitutoyo, Japan), the thickness of two flat glass plates fastened in contact was measured four times to the nearest 0.1 m. This reading was recorded as reading A. The cement for each group (*n* = 10) was mixed according to the manufacturer’s instructions. After mixing, a consistent amount of each cement mixture was then distributed between the two glass plates. A load of 147 N was applied on the upper glass plate using a universal testing machine (Shimadzu Autograph AG–X Plus, Kyoto, Japan). The overall thickness of the plates with the specimen in between was recorded as reading B after seven minutes. The final combined film thickness for the specimen under test was determined as the difference between the thickness of the plates with and without the material between (B-A) [29].

Initial setting times was determined by the Gillmore needles (Humboldt MFG., Norridge, IL, USA) according to ADA specifications [30]. The initial setting time was calculated using a light needle with a tip diameter of 2.12 mm and a weight of 113.4 g. Every 30 s, the needle was positioned on the surface. Initial setting times were determined from the beginning of mixing until the needle did not leave a mark on the surface, respectively. Ten samples were measured for each group [31].

### Testing of specimens

All the specimens were polished to remove any irregularities using 600-grit sandpaper. Specimens were protected with one layer of GIC varnish [32].

### Compressive strength test

Ten cylinder-shaped specimens per group (12 mm in height and 6 mm in diameter) were prepared according to the standard specification for dental glass ionomer cements, ANSI/ADA Specification No. 66 (1989) [33]. The specimens were removed from the molds and stored in an incubator (CBM, S.r.l. Medical Equipment, 2431/V, Cremona, Italy) for 24 h in 95 ± 5% relative humidity at 37 °C. Specimens were loaded in compression at a crosshead speed of 1.0 mm/min in a universal testing machine (Shimadzu Autograph AG–X Plus, Kyoto, Japan) until fracture occurred [34].

### Microhardness test

Ten disc-shaped specimens per group (5 mm in height and 2 mm in diameter) were prepared. The specimens were removed from the molds and incubated for 24 h in a highly humid environment at 37 °C. Surface microhardness for each specimen was determined using a digital Vickers hardness tester (NEXUS 400TM, INNOVATEST, model no. 4503, Maastricht, Netherlands). The indentations were made within 15 s of dwell time at a load of 100 g at 20 × magnificence. The mean surface microhardness value for each specimen was expressed in Vickers hardness numbers (VHN) [35].

### Solubility test

Solubility was investigated using a Teflon mold measuring 7 mm in diameter and 2 mm in thickness [36], to obtain a disc-shaped specimen (*n* = 10). The specimens of each group were stored in a desiccator with silica gel (Merck KGaA, Darmstadt, Germany) for 2 h and then incubated in an incubator at 37 °C for 22 h. Specimens were weighed on a precision analytical balance instrument (Adam equipment 4 digits precision weighing balance, Adam Equipment Inc., Oxford, UK) with an accuracy of 0.001 g to obtain the initial mass (M1) values. Then, the specimens were immersed in a plastic flask containing 25 mL of distilled water, stored for 7 days, and incubated at 37 °C for 7 days. Then, each specimen was removed, dried gently with absorbent paper, and weighed again to get the mass values of the specimens after immersion (M2) [37]. The percentage of solubility was calculated using the equation [38]:

$$(\mathrm{M}1-\mathrm{M}2)/\mathrm{M}1\,\mathrm{ X }\,100\mathrm{\%}$$

where M1 is the initial mass and M2 is the final mass of the specimens. The test was repeated three times [36].

### Antibacterial activity test

Ten-disc specimens for each group measuring 2 mm in height and 4 mm in diameter were prepared. The specimens were stored in an incubator for 24 h at 37 °C. Then antibacterial activity of the specimens was evaluated against *Streptococcus mutans* bacteria. The antibacterial activity was investigated using agar diffusion method [39]. The *Streptococcus mutans* (EMCC 1815) bacterial strains were acquired. The samples were inoculated on Brain Heart Infusion (BHI) agar plates (HiMedia M211, HiMedia Laboratories Pvt. Limited, Mumbai, India) and then incubation at 37 °C for 3 days, the inhibition zone was observed. The total diameter of the inhibition zone in mm for each group was measured.

### Statistical analysis

The statistical analysis was performed using the Statistical Package for the Social Sciences (SPSS) 16.0 statistical software (IBM-SPSS version 27.0, New York, NY, USA). Data was analyzed using independent sample *t*-tests to compare mean values of compressive strength, surface microhardness, solubility, and antimicrobial activity. The significance level was set at *p* ≤ 0.05.

## Results

### Chemical characterization results

The chemical composition of the control and treated specimens analyzed by XRF is represented in Table 1.

**Table 1:** Chemical compositions (mol%) of the treated and untreated GIC specimens by XRF analysis

**Chemical Composition**	**Untreated GIC (control)**	**Treated GIC**
**SiO**_**2**_	24.43	23.9
**Al**_**2**_**O**_**3**_	13.55	13.35
**Fe**_**2**_**O**_**3**_	0.93	0.92
**P**_**2**_**O**_**5**_	6.55	7.1
**CaO**	35.44	34.34
**CaF**_**2**_	15.57	16.2
**SO**_**3**_	2.52	2.66
**K**_**2**_**O**	0.16	0.17
**SrO**	0.26	0.32
**TiO**_**2**_	0.18	0.23
**ZnO**	0.1	0.1
**ZrO**_**2**_	0.23	0.42
**MoO**_**3**_	0.05	0.05
**Nb**_**2**_**O**_**5**_	0.03	0.03
**Ag**	-	0.2
**C**	-	0.01

### Physical characterization results

#### Film thickness

The results of the film thickness of the control and treated specimens are represented in Table 2. There was no significant difference (*P* = 0.07) in the film thickness values between untreated GIC (control) and treated GIC (24 µm and 24.4 µm respectively).

**Table 2:** Descriptive statistics. Mean, Median, Maximum, Minimum, and Standard Deviation values for film thickness test (µm)

	**Mean**	**Median**	**Maximum**	**Minimum**	**Standard Deviation**	***P* value**
**Untreated GIC (control)**	24.04	24.1	24.6	23.3	± 0.371	*P* = 0.07
**Treated GIC**	24.42	24.5	25.1	23.5	± 0.487	

^*^Corresponds to statistically significant difference (*P* ≤ 0.05)

#### Setting time

The results of the initial setting time of the control and treated specimens are represented in Table 3. There was no significant difference (*P* = 0.5) in the initial setting time between untreated GIC (control) and treated GIC (97.7 and 98.7 s respectively).

**Table 3:** Descriptive statistics. Mean, Median, Maximum, Minimum, and Standard Deviation values for initial setting time test (seconds)

	**Mean**	**Median**	**Maximum**	**Minimum**	**Standard Deviation**	***P* value**
**Untreated GIC (control)**	97.7	98	102	94	± 2.86	*P* = 0.5
**Treated GIC**	98.7	99.5	105	92	± 4.16	

^*^Corresponds to statistically significant difference (*P* ≤ 0.05)

#### Testing results

##### Compressive strength results

The results of the compressive strength are represented in Table 4. The compressive strength value of the treated GIC with the Ag-doped CNT group (172.7 MPa) was significantly higher than the untreated GIC (control) group (105 MPa), (*P* = 0.0001*).

**Table 4:** Descriptive statistics. Mean, Median, Maximum, Minimum, and Standard Deviation values for compressive strength (MPa)

	**Mean**	**Median**	**Maximum**	**Minimum**	**Standard Deviation**	***P* value**
**Untreated GIC (control)**	105^a^	105	106	103	± 0.33	*P* = 0.0001^*^
**Treated GIC**	172.7^b^	172.3	175	171	± 0.43	

Different superscript letters (^a,b^) indicate significance

^*^Corresponds to statistically significant difference (*P* ≤ 0.05)

### Microhardness results

The results of the microhardness are represented in Table 5. The Vickers microhardness value of the treated GIC with the Ag-doped CNT group (92.5 VHN) was significantly higher than the untreated GIC (control) group (61.3 VHN), (*P* = 0.0001*).

**Table 5:** Descriptive statistics. Mean, Median, Maximum, Minimum, and Standard Deviation values for microhardness (VHN)

	**Mean**	**Median**	**Maximum**	**Minimum**	**Standard Deviation**	***P* value**
**Untreated GIC (control)**	61.3^a^	61.3	62	60	± 0.72	*P* = 0.0001^*^
**Treated GIC**	92.5^b^	92.4	94	91.5	± 0.81	

Different superscript letters (^a,b^) indicate significance

^*^Corresponds to statistically significant difference (*P* ≤ 0.05)

### Solubility results

The results of the solubility are represented in Table 6. There was no significant difference in solubility mean values among the control group (3.4%) and treated group (3.1%), (*P* = 0.1).

**Table 6:** Descriptive statistics. Mean, Median, Maximum, Minimum, and Standard Deviation values for solubility (%)

	**Mean**	**Median**	**Maximum**	**Minimum**	**Standard Deviation**	***P* value**
**Untreated GIC (control)**	3.4	3.2	4	2.9	± 0.32	*P* = 0.1
**Treated GIC**	3.1	3.0	4.2	2.4	± 0.44	

^*^Corresponds to statistically significant difference (*P* ≤ 0.05)

### Antibacterial activity test

The results of the agar diffusion test against *Streptococcus mutans* are represented in Table 7. There was a significant difference in the antibacterial activity against *Streptococcus mutans* in both groups. The control group showed a significantly lower inhibition zone value around the discs (1 mm). While the treated group showed a significantly higher inhibition zone value around the peripheries of the treated discs (9 mm), (*P* = 0.0001*).

**Table 7:** Descriptive statistics. Mean, Median, Maximum, Minimum, and Standard Deviation values for *Streptococcus mutans* inhibition zone after 3 days (mm)

	**Mean**	**Median**	**Maximum**	**Minimum**	**Standard Deviation**	***P* value**
**Untreated GIC (control)**	1^a^	1	1.2	0.8	± 0.13	*P* = 0.0001*
**Treated GIC**	9^b^	9	9.3	8.8	± 0.14	

Different superscript letters (^a,b^) indicate significance

^*^Corresponds to statistically significant difference (*P* ≤ 0.05)

## Discussion

Conventional GICs are extensively used in dentistry because of their distinctive qualities, such as chemical bonding to tooth structures, necessitating the least amount of dental preparation [40], chemical bonding with base metals, thermal compatibility with enamel, biocompatibility and low cytotoxicity, fluoride release ability, and provide chemical bonding to tooth structure, thus requiring a minimum tooth preparation [40]. However, one of the main issues with GIC is that it has a lower mechanical properties than recent resin composite materials [41]. The durability of GIC is affected by numerous factors, such as microhardness and solubility [36]. Solubility is one of the most crucial factors for evaluating the quality of materials used for liners, bases, luting agents, and restorations [36]. The solubility of restorative materials has a great influence on their stability, biocompatibility, and longevity [36].

As secondary caries is one of the most prevalent causes of dental restoration failure, it has also frequently been noted that the presence of biofilm on the surface of the restorative materials may facilitate the emergence of secondary caries lesions [42]. Secondary caries is mainly developed by the invasion of cariogenic bacteria, principally *Streptococcus mutans* [43]*.* Therefore, the antibacterial activity of restorative materials is of great value to avoid the possibility of secondary caries along the tooth-restoration interface [44]. The ideal’ dental cement and restoration should have several features, such as high surface and mechanical characteristics, adequate setting time, and a low film thickness (less than 25 µm) for the luting agent [45].

The development of nanotechnology has led to the development of new caries management strategies. In low concentrations, Ag nanoparticles have substantial antibacterial activity against *Streptococcus mutans* [46]. Ag nanoparticles may exhibit a synergistic antibacterial effect when combined with other antibacterial element [47].

The exceptional mechanical characteristics of CNT make it possible to be incorporated into dental materials for a pioneering functional application [48]. Ag-nanoparticles and CNTs could be functionalized together to create novel fillers that could be able to enhance the mechanical properties, surface microhardness, solubility, and antimicrobial activity of conventional GIC [24]. The functionalized CNT fillers were incorporated in a minimal concentration of 0.01 wt.% in an attempt to improve the mechanical properties without deterioration of the surface microhardness [49].

A compressive strength test simulates the load exerted on materials used in dental practice, the majority of mastication stresses are compressive in nature [50]. Compressive tests represent the critical value at which the material can withstand during the masticatory process [51]. The posterior teeth can withhold masticatory forces up to 125 MPa, while the primary dentition can only withstand compressive strength up to 100 MPa [52].

The current study has been carried out to evaluate the effect of incorporating 0.01 wt.% Ag-doped CNT fillers into commercially available conventional GIC on compressive strength, surface microhardness, solubility, and antimicrobial activity against *Streptococcus* mutans. The null hypothesis was rejected, where the incorporation of 0.01 wt.% Ag-doped CNT into conventional GIC significantly affected the compressive strength, surface microhardness and antimicrobial activity against *Streptococcus* mutans compared to the control group.

The result of the XRF confirmed that the chemical compounds of the Ag-doped CNTs were incorporated into glass ionomer material specimens. In accordance with ADA No. 8 [53], dental cement should have a film thickness no more than 25 µm for water-based luting cements and no more than 50 µm for resin-based cements. Minimal film thickness will lead to enhanced restoration retention and reduce marginal discrepancies [54]. Both treated and control groups provide a film thickness lower than 25 µm. Moreover, the results revealed no significant difference between groups, which may be attributed to the lower concentration of the added fillers in the treated groups. As regard the initial setting time, the results revealed no significant difference between groups, which may also be attributed to the lower concentration of the added fillers in the treated groups, which leads to a negligible effect.

The results of the present study showed that the addition of 0.01 wt.% Ag-doped CNT fillers into dental GIC significantly improved the compressive strength. This finding may be attributed to the expected reinforcing effect of Ag-doped CNT fillers [17]. Moreover, the incorporation of the minute amount of the strong CNT nanofillers may be responsible for inhibiting crack propagation by transferring the stress from the weaker matrix to the stronger nanoparticle fillers without deterioration of the interfacial bonding [55]. These results are in accordance with previous studies that showed a reinforcement ability for dental restorations after incorporation of CNT [56]. However, these results disagree with the study conducted by Spinola et al., which reported that the incorporation of 1 wt.% CNT fillers had a negative effect on the compressive strength of glass ionomers, which may be due to the non-homogeneous distribution of the nanoparticle fillers and the consequent formation of voids [57].

Moreover, The increase in surface microhardness in the treaed groups can be referred to the homogenous dispersion of hard Ag-doped CNT filler nanoparticles within the matrix [58]. Both the control and modified groups exhibited similar solubility percentages; this finding may be explained by the small amount of Ag-doped CNT nanoparticle fillers added, which was not enough to improve the dissolution of the matrix. Furthermore, the incorporation of Ag-doped CNT nanoparticle fillers improved the antibacterial effect of the treated groups. This finding may be due to the fact that Ag nanoparticles displayed powerful bactericidal activity against *Streptococcus mutans* by rupturing their outer cellular membrane [59, 60]. Additionally, multi-walled CNT exhibit potent antibacterial properties and have the ability to penetrate bacterial cell walls [61]. Moreover, other aspects could have a significant impact on the mechanical characteristics of GICS, such as the depth of cure [62, 63]. Therefore, future studies are needed, taking into careful account also these variables.

The present study's limitation is that the experimental conditions did not completely replicate clinical ones. However, all the specimens were subjected to the same testing conditions. Further studies are recommended to assess the effect of the incorporation of Ag-doped CNT to GIC in different concentrations in order to recognize the optimum concentrations required to improve the mechanical properties. Moreover, it is recommended to investigate the color changes associated with each concentration.

## Conclusions

The innovatively reinforced GIC with 0.01 wt.% Ag doped CNT fillers had the opportunity to be used as an alternative to conventional GIC dental cements as it provides enhanced compressive strength, surface microhardness, and anti-bacterial activity against *Streptococcus mutans* compared to the conventional GIC.

## Data Availability

The datasets generated during and/or analyzed during the current study are not publicly available due to institutional policy but are available from the corresponding author on reasonable request.

## Abbreviations

GICs: Glass ionomer cements
CNTs: Carbon nanotubes
Ag: Silver
wt.%: weight percent
MREC: Medical Research Ethical Committee
NRC: National Research Centre
XRF: X-ray fluorescence
VHN: Vickers microhardness number
EMCC: The Egypt Microbial Culture Collection
BHI: Brain Heart Infusion
MPa: Megapascal
